# A practical work around for breast density distribution discrepancies between mammographic images from different vendors

**DOI:** 10.1007/s00330-025-11383-w

**Published:** 2025-01-31

**Authors:** Tobias Wagner, Lesley Cockmartin, Yao-Kuan Wang, Nicholas Marshall, Hilde Bosmans

**Affiliations:** 1 Department of Imaging and Pathology, Division of Medical Physics & Quality Assessment, KU Leuven, Leuven, Belgium; 2 Department of Imaging and Pathology, Division of Medical Physics & Quality Assessment, UZ Leuven, Leuven, Belgium

**Keywords:** Mammography, Breast neoplasms, Breast density, Early detection of cancer

## Abstract

**Objectives:**

Investigate the impact of mammography device grouped by vendor on volumetric breast density and propose a method that mitigates biases when determining the proportion of high-density women.

**Materials and methods:**

Density grade class and volumetric breast density distributions were obtained from mammographic images from three different vendor devices in different centers using breast density evaluation software in a retrospective study. Density distributions were compared across devices with a Mann–Whitney U test and breast density thresholds corresponding to distribution percentiles calculated. A method of matching density percentiles is proposed to determine women at potentially high risk while mitigating possible bias due to the device used for screening.

**Results:**

2083 (mean age 59 ± 5.4), 531 (mean age 58.8 ± 5.7) and 244 (mean age 60.7 ± 6.0) screened women were evaluated on three vendor devices, respectively. Both the density grade distribution and the volumetric breast density were different between Vendor 1 and Vendor 2 data (*p* < 0.001) and between Vendor 1 and Vendor 3 data (*p* < 0.001). Between Vendor 2 and Vendor 3, no significant difference was observed (*p* = 0.67 for density grade, *p* = 0.29 for volumetric density). To recruit the top 10% of women with extremely dense breasts required respective density thresholds of 16.1%, 13.6% and 13.8% for the three vendor devices.

**Conclusion:**

Density grade class and volumetric breast density distributions differ between devices grouped by vendor and can result in statistically different breast density distributions. Percentile-dependent density thresholds can ensure unbiased selection of high-risk women.

**Key Points:**

***Question*** *Does the use of x-ray systems from different vendors influence breast density evaluation and the resulting selection of high-risk women during breast cancer screening?*

***Findings*** *Statistically significant differences were observed between breast density distributions of different vendors; a method of matching via percentiles is proposed to prevent biased density evaluations.*

***Clinical relevance*** *Measured breast density distributions differed between X-ray devices. A workaround is proposed that determines density thresholds corresponding to a specified population, allowing the same proportion of women to be selected with a density algorithm.*

**Graphical Abstract:**

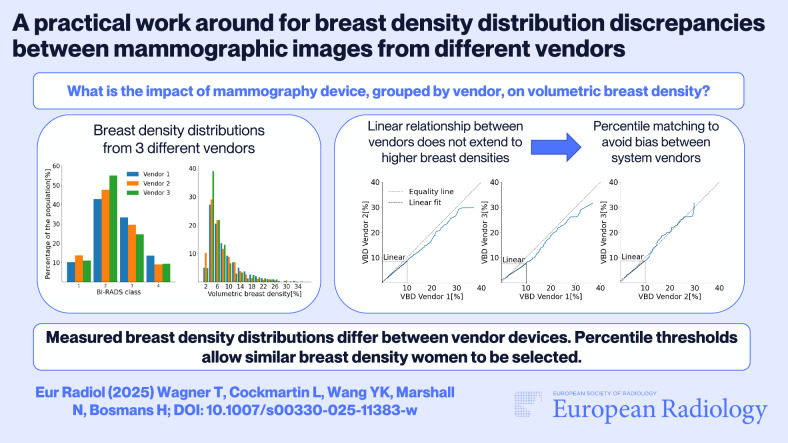

## Introduction

Demand for risk-based [1–4] breast cancer screening has increased in recent years. While the possibility of improving screening performance is appealing, a comprehensive method of selecting women at high risk is not available in the literature [4]. In addition to the current research focus on AI methods [5], breast density is also recognized as an independent risk factor for breast cancer and is being adopted in epidemiological models [6]. Following the guidelines of the American College of Radiology’s Breast Imaging Reporting and Data System (BI-RADS) 5th Edition [7], women can be assigned to one of four different BI-RADS classes ranging from almost entirely fatty to extremely dense breasts. Since the introduction of the 5th Edition BI-RADS, the correlation between the volumetric breast density and the corresponding BI-RADS classification also considers the masking effect [8, 9]. This change resulted in the transition from the 4th to the 5th edition of the BI-RADS assessment guidelines [8, 9].

BI-RADS class 4 covers very dense breasts with masking effect and is of particular interest when investigating breast cancer risk [10]. Whether only women in BI-RADS 4 category should be invited for supplemental screening is currently not known. In addition to a visual and inevitably subjective assessment of breast density, more objective image-based calculations of breast density can be performed with the help of automated computer-based density measurements [11]. This calculation involves separating fibroglandular and fatty tissue, which generate different pixel values based on their distinct attenuation characteristics. Device-related factors, including acquisition and system parameters, are known to impact the resulting image quality [12]. Ensuring a vendor-neutral breast density estimate is crucial to prevent an inherent bias in assigning high-risk status during screening, which would affect the number of women invited for an adapted screening regime. While previous studies have investigated breast density algorithms [13, 14], comprehensive research on the impact of mammography device on density estimates remains limited [15], emphasizing the need for methods that enable a normalized selection of certain density populations within the screened population.

This study proposes a simple method to reduce bias in breast density selection made using images from devices of different vendors. The densities corresponding to particular percentiles were computed from the breast density distributions, and these were found independently for each vendor.

## Materials and methods

### Dataset

The use of retrospective data in this study was approved by the local ethical committee. A total of 2154, 540, and 255 exams were analyzed from respectively Siemens Mammomat Inspiration (Siemens Healthineers), GE Senographe Essential (GE HealthCare), and Hologic Selenia Dimensions (Hologic Inc.) mammography devices. These images were from women who were eligible for the Flemish breast cancer screening program and were available in DICOM ‘FOR PROCESSING’ format. It is assumed that the underlying characteristics of the population screened on different vendors are homogeneous. The study used successive screening examinations collected during the year 2017 at three screening centers in the region of Flanders, Belgium. Diagnostic mammograms as well as exams with missing breast density values, which occasionally occur for very small breasts, were excluded from the study.

### Determining volumetric breast density and BI-RADS class

Volumetric breast density and BI-RADS density class were estimated using TruDensity® by Volpara Health. For data from GE and Hologic, two software versions, namely 1.5.2.1 and 1.5.4, were used depending on the timing of data collection and analysis. For Siemens data, both versions were applied with a mean absolute difference in volumetric breast density between both software versions on a dataset of 2114 Siemens cases of 0.014. We proceeded with the most recent 1.5.4 software version results for the Siemens data in this study. Volpara Density Grade^TM^ (VDG) is divided into four categories, from 1 to 4, and designed to correlate with the BI-RADS 5th edition classes of A, B, C and D. Correlation between the VDG and BI-RADS 5th Ed has been shown in earlier studies [16]. Volumetric percentage of breast density is the ratio of the volume of the fibroglandular dense tissue within the breast to the total breast volume. Volumetric percentages were averaged across all four mammographic views in this study. The VDG categories take both the volumetric density and the masking effect into account when assigning the mammographic image to one of four classes. The final VDG for the patient was determined by the VDG category of the view with the highest class.

To gain a better understanding of the impact of masking effect and breast density on the VDG classes, volumetric densities were compared between the different classes with a frequency histogram. Furthermore, for each VDG group, the upper and lower densities were measured corresponding to 95% of cases.

### Statistical comparison of density distributions

The Mann–Whitney U test [17] was used to establish whether there were statistically significant differences between the breast density distributions. The null hypothesis tested is that for two random samples drawn from two populations, the probability of one being greater than the other is the same as the probability of it being smaller. Both the VDG classes and the volumetric breast density distributions between vendors were analyzed.

This is a nonparametric test that does not require specific knowledge or assumptions about the underlying distributions. The null hypothesis is identical for both VDG class and volumetric breast density comparisons. A significance level α of 0.05 was used.

### Density thresholds to select the same proportion of women with extremely dense breasts

To deal with discrepancies between volumetric breast density distributions, a method is needed to select the same proportion of women in the high breast density group, regardless of the device used for their examination. Assuming that the underlying demographic parameter distribution of women participating in screening in different cities in Flanders is the same, partitioning the respective distributions into quantiles yields density values for different percentiles in the distribution. These breast density values corresponding to specific percentiles (‘density thresholds’) were calculated for images acquired on devices from the different vendors. In the context of risk stratification, the 90^th^ percentile could be used; this breast density threshold would separate the breasts with the highest 10% density values from the rest. This calculation could be repeated for other percentiles to satisfy local procedures in place.

The vendor-specific contributions were also compared across all percentiles by means of a quantile-quantile plot [18], which showed whether systematic differences between vendors were present. In such a case, a linear mapping of density values from one vendor to another vendor may be possible.

## Results

### Dataset

**Fig. 1:**
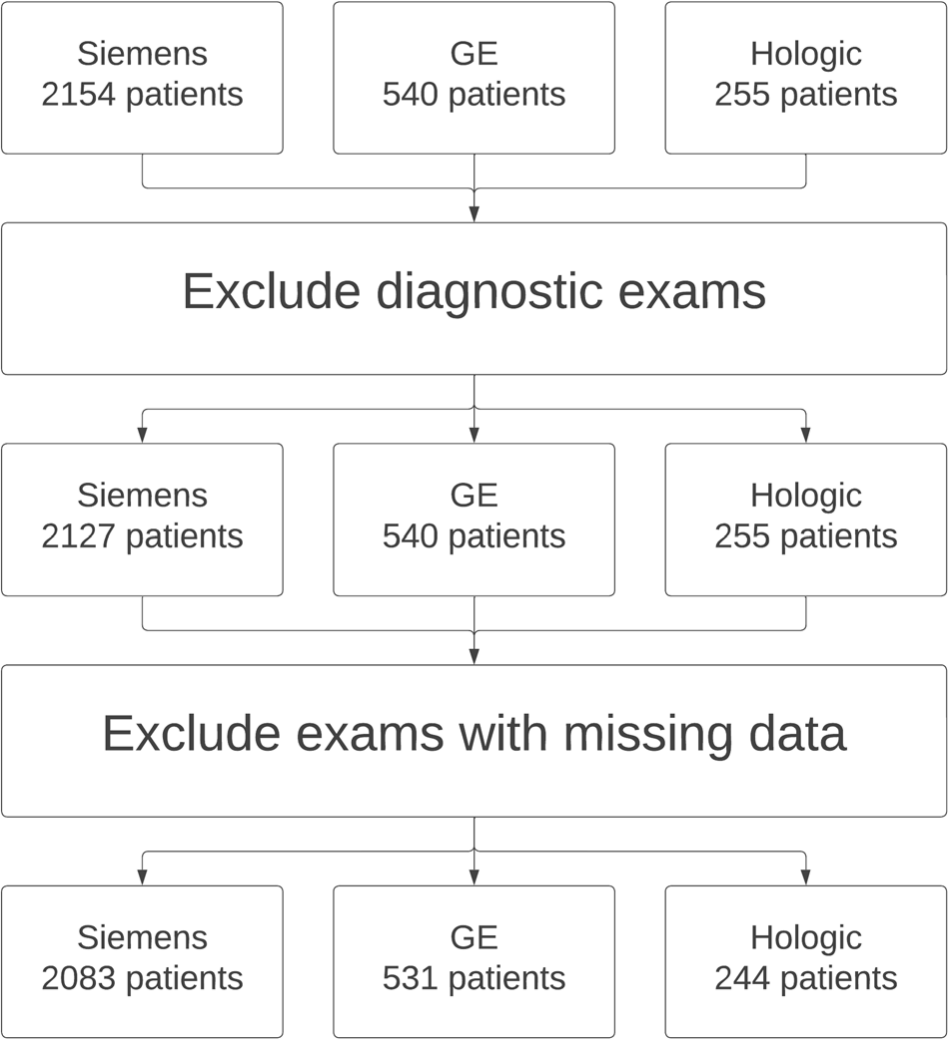
Flowchart of the data exclusion process. Diagnostic exams as well as exams with missing image data were excluded from the study

After application of the exclusion criteria to the full dataset, 2083, 531 and 244 exams were evaluated for Siemens, GE and Hologic, respectively. A flowchart of the exclusion process is shown in Fig. 1.

The demographic and breast density information of the dataset used in this study can be found in Table 1.

**Table 1:** Demographics of the dataset used in this study

Characteristics	Siemens	GE	Hologic
All examinations	2083 (100%)	531 (100%)	244 (100%)
Age			
< 50	62 (3.0%)	14 (2.6%)	8 (3.3%)
50–60	1075 (51.6%)	278 (52.4%)	81 (33.2%)
60–70	946 (45.4%)	239 (45.0%)	155 (63.5%)
Mean ± Std	59.0 ± 5.4	58.8 ± 5.7	60.7 ± 6.0
VDG class			
1	207 (9.9%)	73 (13.7%)	27 (11.1%)
2	897 (43.1%)	253 (47.6%)	134 (54.9%)
3	700 (33.6%)	157 (29.6%)	60 (24.6%)
4	279 (13.4%)	48 (9.0%)	23 (9.4%)

For each demographic, absolute and relative number of examinations are displayed. The Volpara Density Grade^TM^ (VDG) class depicts the values calculated by TruDensity®

### Determining volumetric breast density and VDG class

**Fig. 2:**
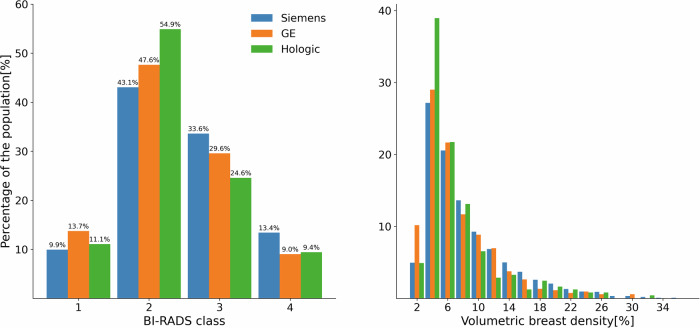
Breast density distributions for the different vendors. The Volpara Density Grade^TM^ (VDG) class distribution can be seen on the left, while the volumetric breast density distribution is depicted on the right

The VDG class and volumetric breast density distributions for the different vendors can be seen in Fig. 2. It is apparent that there is some discrepancy between the proportion of very dense cases. For GE and Hologic devices, this is 9.0% and 9.4%, respectively, while that for Siemens is 13.4%. The volumetric breast density distribution for all three vendors reveals a heavily right-tailed distribution of the values, leading to a lower number of samples in the high-density area.

**Fig. 3:**
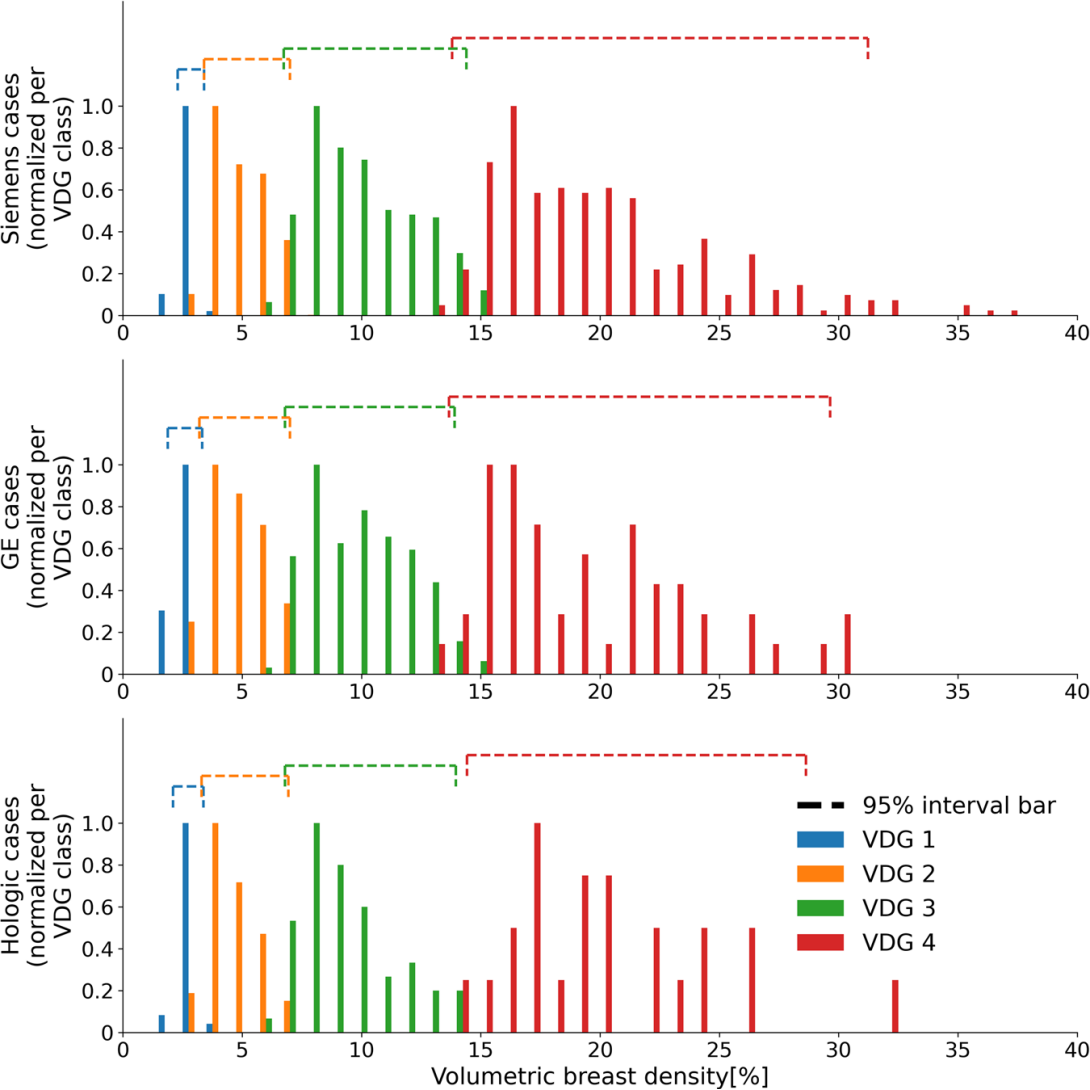
Normalized volumetric breast density distribution for each Volpara Density Grade^TM^ class for the different vendors. The values are normalized for each VDG class. The horizontal bars depict the range of volumetric densities that 95% of values fall into for each class

The frequency histogram of volumetric breast density of each VDG class for all vendors is shown in Fig. 3. Along with the volumetric breast densities, the 95% interval bar depicts the density range that contains 95% of all the data. Despite the masking effect contribution to the categorization process within the VDG class system, these 95% interval bars separate the four different VDG classes with little overlap.

### Statistical comparison of density distributions

The results of the Mann–Whitney U test applied to the dataset, as well as accompanying *p*-values, are displayed in Table 2.

**Table 2:** Mann–Whitney U test results

Density description	Comparison	Mann–Whitney U test
	Vendor 1 (Cases) vs. Vendor 2 (Cases)	*p*-value
VDG class	Siemens (2083) vs. GE (531)	< 0.0001
	Siemens (2083) vs. Hologic (244)	0.0007
	GE (531) vs. Hologic (244)	0.67
Volumetric breast density	Siemens (2083) vs. GE (531)	< 0.0001
	Siemens (2083) vs. Hologic (244)	< 0.0001
	GE (531) vs. Hologic (244)	0.29

Both Volpara Density Grade^TM^ (VDG) class and volumetric breast density distributions were compared between vendors. The result is given as a *p*-value

The analysis of the VDG class distributions shows a statistically significant difference between the Siemens and GE data (*p* < 0.001) and the Siemens and Hologic data (*p* < 0.001). However, there is no statistically significant difference between GE and Hologic distributions (*p* = 0.67).

The volumetric breast density data show similar results, with statistically significant differences between the Siemens and GE distributions (*p* < 0.001) and for the distributions of the Siemens and Hologic devices (*p* < 0.001), while the GE and Hologic devices are closer to each other (*p* = 0.29). An analysis of the effect of varying dataset size on the resulting *p*-value of the Mann–Whitney U test for the different vendors can be found in Tables S1 and S2 of the Supplementary Material.

### Density thresholds to select the same proportion of women with extremely dense breasts

The density thresholds corresponding to different percentiles are shown in Table 3. Due to the heavily right-tailed volumetric breast density distribution, the confidence interval of the density thresholds becomes wider at higher densities. An analysis of the effect of sample size on density thresholds can be found in Figure S1 in the Supplementary Materials.

**Table 3:** Density thresholds for different percentiles for the different vendors

		Density threshold	
Percentile	Siemens	GE	Hologic
10	3.4 [3.3, 3.5]	3.0 [2.8, 3.2]	3.2 [3.1, 3.4]
20	4.0 [3.9, 4.2]	3.6 [3.4, 3.8]	3.6 [3.5, 3.8]
30	4.8 [4.6, 4.9]	4.3 [4.0, 4.6]	4.1 [3.8, 4.4]
40	5.7 [5.5, 5.9]	5.0 [4.7, 5.3]	4.7 [4.4, 5.1]
50	6.7 [6.4, 6.9]	5.8 [5.5, 6.2]	5.3 [4.9, 5.8]
60	7.9 [7.6, 8.3]	6.8 [6.4, 7.3]	6.1 [5.6, 7.0]
70	9.7 [9.3, 10.0]	8.3 [7.6, 9.1]	7.5 [6.6, 8.4]
80	12.3 [11.8, 12.7]	10.6 [9.8, 11.4]	9.2 [8.4, 10.4]
90	16.1 [15.5, 17.0]	13.6 [12.5, 15.0]	13.8 [11.1, 17.1]

Density thresholds with [95% confidence interval] as a function of percentile

**Fig. 4:**
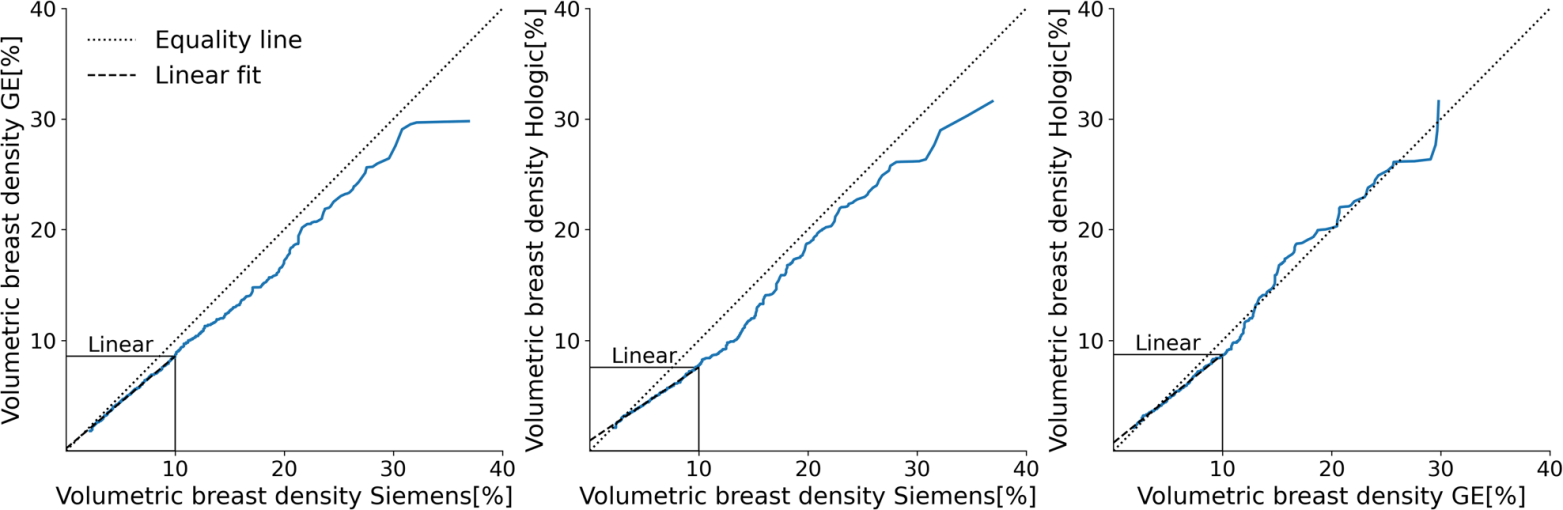
Quantile-quantile plots for all vendor pairs. Distributions are matched by percentiles and plotted against each other. The equality line (dotted) represents equal percentiles. For low volumetric breast density values, a linear fit (dashed) can be used to describe the data

Figure 4 plots the quantile-quantile plots between the different vendors. A common feature seen in these distributions is the linear relationship between any two vendors for the lower volumetric breast densities. This suggests that calibration by linear mapping would be sufficient to align the distributions at this lower density; however, this would not apply to higher volumetric densities, where the relationship between vendors becomes non-linear. From the volumetric breast density distributions shown in Fig. 2, most of the breast density values fall into the linear regime; however, if risk stratification is required, where the highest density group carries significant weight, this linear approach falls short.

## Discussion

Breast density is one of the parameters proposed for use in risk-stratified breast screening [19–21]. Despite the contribution of the masking effect on the BI-RADS classification, whose exact calculation is proprietary information only available to Volpara, volumetric breast density values can be used to distinguish between the different classes. For both VDG class as well as volumetric breast density distributions, this study showed a significant difference between mammography vendors when using the TruDensity® algorithm from Volpara Health. While these differences can mostly be accounted for with a linear mapping at lower densities, this does not extend to higher densities, which are important in identifying women with a high risk of breast cancer. It is possible that differences in the imaging system (e.g., x-ray spectrum and detector) are impacting the distributions; however, Volpara states that TruDensity is independent of device type. Both the Siemens and Hologic devices employ similar technology (detector and x-ray spectrum), and therefore, if the imaging chain is impacting the calculated density, we might have found similar density distributions for the Siemens and Hologic devices. However, this was not the case, suggesting that factors other than detector and source are at play. In a study sampling 20 women, Damases et al [15] also found no significant difference between density measurements of GE and Hologic devices and concluded that mammography system does not affect measured breast density. The current study has shown a statistically different density distribution for the Siemens device compared to the GE and Hologic systems, with data from between 244 and 2083 women. Supplemental testing has proven that these results are independent of the sample size differences. Finally, to avoid potential bias due to imaging device when selecting high-risk women, percentile-dependent density thresholds can be used to partition a population into different risk groups.

One fundamental assumption of this work is that the underlying populations measured on the different vendors are the same. A previous study by Ohmaru et al [22] investigating age-related change in mammographic density shows little change in BI-RADS class distribution for women after the age of 50 years. Another consideration is the influence of ethnicity on breast density, which has been investigated by McCormack et al [23], finding significant differences between Caucasians and Afro-Caribbeans as well as between Caucasians and South Asians. However, for the present study, the data from the different vendors was taken from three different sites that are within 50 km of each other, all of which are located outside large urban centers. Data show that 11% of people in Flanders in 2023 have a foreign nationality, and six out of ten foreigners originate in one of the 27 countries of the European Union [24].

There are some limitations to this study. It would be useful to include additional vendors in the analysis. Increasing the number of women, especially in the high-density range, would reduce the range of the 95% confidence interval of density thresholds.

In conclusion, remedying the influence of different imaging devices on breast density evaluations is crucial for mitigating biases in breast density-based risk stratification.

## Supplementary information

Supplementary Material

## Abbreviations

BI-RADS: Breast Imaging Reporting and Data System
VDG: Volpara Density Grade^TM^
