# Supplementary material for: A practical work around for breast density distribution discrepancies between mammographic images from different vendors
Source: Eur Radiol. 2025 Jan 31;35(8):4885–92. doi: 10.1007/s00330-025-11383-w (PMC12226699; doi:10.1007/s00330-025-11383-w)
Supplement: Supplementary file 1 — Supplementary Material [file 330_2025_11383_MOESM1_ESM.docx]

Supplementary material

### **Influence of population size**

In order to examine the influence of the number of cases, the dataset distributions for the 3 vendors were resampled using Inverse Transform Sampling. The sample sizes were chosen to represent the population sizes of the respective datasets (244, 531, 2083). For each sample size, the respective sampled populations were compared using the Mann-Whitney U test. If, -for a given pair of sampled distributions, -the p-value is below 0.05, then a 0 was recorded, otherwise a 1 was recorded. The experiment was repeated 10,000 times, after which the average of the recorded number was calculated, which quantifies the proportion of p-values below the significance level of 0.05 for the null hypothesis that for two random samples drawn from two populations, the probability of one being greater than the other is the same as the probability of it being smaller. For completeness, two populations drawn from the same vendor were also compared. The analysis was done for distributions of both VDG and volumetric density.

The results are presented in Tables S1 and S2.

*Table S1:* ***Dependence of p-value on sample size for comparison of VDG distributions.*** *For 10,000 samples, the proportion of p-values below the significance level of 0.05 of the Mann-Whitney U test is presented. The* ***X*** *indicates repeated cases.*

|  |  | **Proportion of p-values below significance level of 0.05** | | |
| --- | --- | --- | --- | --- |
|  | **Number of cases resampled** | **Siemens** | **GE** | **Hologic** |
|  | 244 | 0.95 | 0.95 | 0.26 |
| **Siemens** | 531 | 0.95 | 0.11 | 0.03 |
|  | 2083 | 0.95 | 0.00 | 0.00 |
|  | 244 | X | 0.95 | 0.93 |
| **GE** | 531 | X | 0.95 | 0.92 |
|  | 2083 | X | 0.95 | 0.82 |
|  | 244 | X | X | 0.96 |
| **Hologic** | 531 | X | X | 0.95 |
|  | 2083 | X | X | 0.95 |

*Table S2:* ***Dependence of p-value on sample size for comparison of volumetric breast density distributions.*** *For 10,000 samples, the proportion of p-values below the significance level of 0.05 of the Mann-Whitney U test is presented. The* ***X*** *indicates repeated cases.*

|  |  | **Proportion of p-values below significance level of 0.05** | | |
| --- | --- | --- | --- | --- |
|  | **Number of cases resampled** | **Siemens** | **GE** | **Hologic** |
|  | 244 | 0.95 | 0.34 | 0.08 |
| **Siemens** | 531 | 0.95 | 0.06 | 0.00 |
|  | 2083 | 0.95 | 0.00 | 0.00 |
|  | 244 | X | 0.95 | 0.86 |
| **GE** | 531 | X | 0.95 | 0.74 |
|  | 2083 | X | 0.95 | 0.24 |
|  | 244 | X | X | 0.96 |
| **Hologic** | 531 | X | X | 0.95 |
|  | 2083 | X | X | 0.95 |

For both types of distributions, two randomly drawn populations from the same vendor are not statistically different in around 95% of cases, regardless of the chosen sample size. When comparing different vendors, higher sample size results in fewer cases where the distributions are not statistically different. Nonetheless, the proportion of p-values below significance level of 0.05 between GE and Hologic was found to be consistently higher than for Siemens and GE and Siemens and Hologic.

There is some variation in the distribution of p-values but the results of the comparison between the original datasets of the three vendors remained unchanged. A significant difference was found between Siemens and both the GE and Hologic distributions; the GE and Hologic density distributions display a higher degree of similarity.

### **Analysis of sample size effect on percentile thresholds**

The relationship between the sample size and the confidence interval of the 90^th^ percentile density threshold is complex, especially considering that the volumetric breast density distribution does not follow a normal distribution. Therefore, to evaluate the accuracy of the method, the effect of sample size on the uncertainty of the density thresholds at the 90^th^ percentile was examined. Different population sizes were sampled from the underlying volumetric breast density distributions using inverse transform sampling. This method uses a reference cumulative distribution function to sample values from continuous distributions, enabling values to be sampled across the entire range. For the reference distributions, the data from the different vendors were used.

If N is a given sample population size, N values were sampled from the distribution of a given vendor using bootstrapping. This formed the sample population and the 90^th^ percentile density threshold was calculated for this population. Next, the percentiles in the original distribution that correspond to the density thresholds of all sampled populations were obtained and the 95% confidence interval of these values was calculated. Density thresholds were calculated for 100.000 cases and for 50 population sizes logarithmically spaced between 10 and 5000; this was done for each vendor.

For a given population size, 100,000 samples were drawn from the vendor reference distribution with bootstrapping. For each sampled population, the density thresholds of the 90^th^ percentile were calculated. Next, the percentiles that these density thresholds would correspond to in the original distribution were determined. The range of the 95% confidence interval of these percentiles was calculated; the results are depicted in Figure S1.

For Siemens devices, taking into account the dataset size of 2083, the range of the 95% confidence interval of the percentile values is 2.52. For the 531 GE cases, the range is 5.13. For the 244 Hologic cases, the range is 7.60.


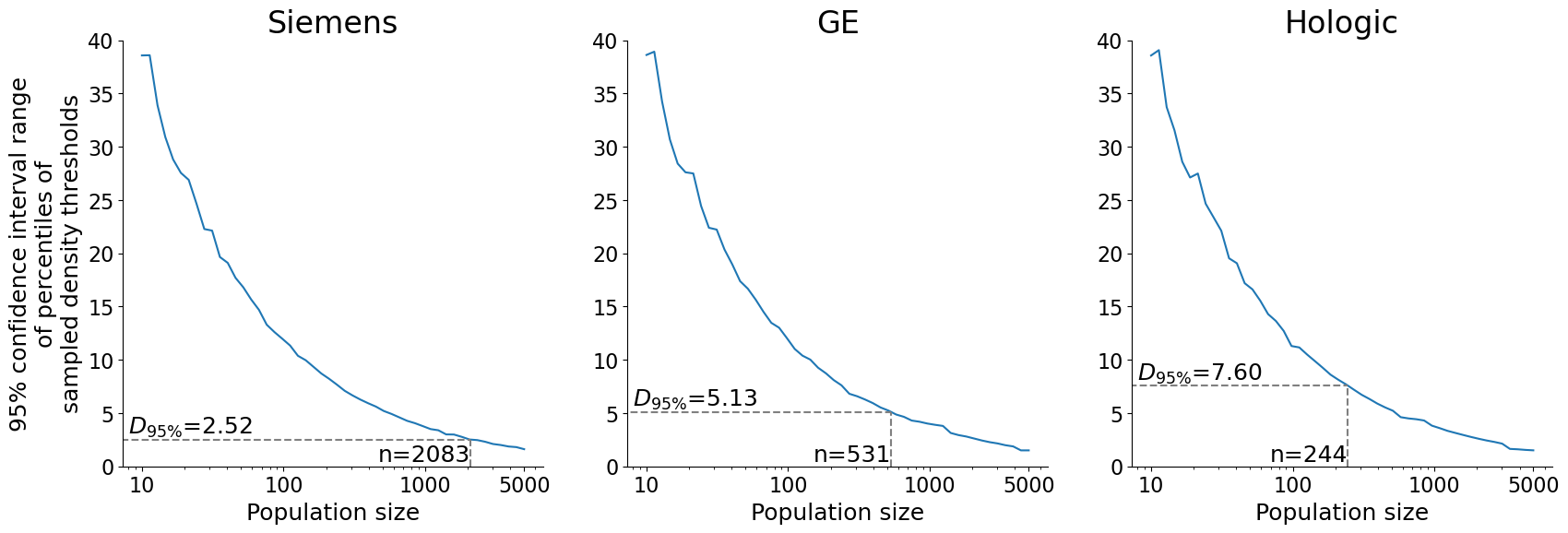


*Figure S1:* ***Sample size analysis for different population sizes.*** *For each population size, the range of the 95% confidence interval of the percentiles corresponding to sampled density thresholds(D_95%_) is depicted on the y-axis. The numbers displayed next to the dashed line denote the range of the 95% confidence interval of the percentiles for the population sizes of the datasets of the respective vendors.*
